# A 5-year natural history study in LAMA2-related muscular dystrophy and SELENON-related myopathy: the Extended LAST STRONG study

**DOI:** 10.1186/s12883-024-03852-4

**Published:** 2024-10-23

**Authors:** E. C. M. de Laat, S.L.S. Houwen- van Opstal, K. Bouman, J. L. M. van Doorn, D. Cameron, N. van Alfen, A. T. M. Dittrich, E. J. Kamsteeg, H. J. M. Smeets, J. T. Groothuis, C. E. Erasmus, Nicol C. Voermans

**Affiliations:** 1 Department of Neurology, Donders Institute for Brain, Cognition and Behaviour, Radboud University Medical Center, Nijmegen, The Netherlands; 2 Department of Rehabilitation, Donders Institute for Brain, Cognition and Behaviour, Radboud University Medical Center, Amalia Children’s Hospital, Nijmegen, The Netherlands; 3 Department of Neurology, Clinical Neuromuscular Imaging Group, Donders Institute for Brain, Cognition and Behaviour, Radboud University Medical Center, Nijmegen, The Netherlands; 4 Department of Radiology, Clinical Neuromuscular Imaging Group, Radboud University Medical Center, Nijmegen, The Netherlands; 5 Department of Pediatrics, Radboud University Medical Center, Radboud Institute for Health Sciences, Amalia Children’s Hospital, Nijmegen, The Netherlands; 6 Department of Human Genetics, Radboud University Medical Center, Nijmegen, The Netherlands; 7 Department of Toxicogenomics, Research Institutes MHeNS and GROW, Maastricht University, Maastricht, The Netherlands; 8 Department of Pediatric Neurology, Donders Institute for Brain, Cognition and Behaviour, Amalia Children’s Hospital, Radboud University Medical Center, Nijmegen, The Netherlands

**Keywords:** SELENON, SEPN1, LAMA2, Merosin-deficient congenital muscular dystrophy type 1A (MDC1A), Natural history, Follow-up, Outcome measures, Trial readiness

## Abstract

**Background:**

SELENON-related myopathy (SELENON-RM) is a rare congenital myopathy characterized by slowly progressive axial muscle weakness, rigidity of the spine, scoliosis, and respiratory insufficiency. Laminin-a2-related muscular dystrophy (LAMA2-MD) has a similar clinical phenotype, which ranges from severe, early-onset congenital muscular dystrophy type 1A (MDC1A) to milder forms presenting as childhood- or adult-onset limb-girdle type muscular dystrophy. The first 1.5-year natural history follow-up showed that 90% of the patients had low bone quality, respiratory impairments were found in all SELENON-RM and most of the LAMA2-MD patients, and many had cardiac risk factors. However, further extensive knowledge on long-term natural history data, and clinical and functional outcome measures is needed to reach trial readiness. Therefore, we extended the natural history study with 3- and 5-year follow-up visits (Extended LAST STRONG).

**Methods:**

The Extended LAST STRONG is a long-term natural history study in Dutch-speaking patients of all ages diagnosed with genetically confirmed SELENON-RM or LAMA2-MD, starting in September 2023. Patients visit our hospital twice over a period of 2 years to complete a 5-year follow up from the initial LAST-STRONG study. At both visits, they undergo standardized neurological examination, hand-held dynamometry (age ≥ 5 years), functional measurements, muscle ultrasound, respiratory assessments (spirometry, maximal inspiratory and expiratory pressure, sniff nasal inspiratory pressure; age ≥ 5 years), Dual-energy X-ray absorptiometry (DEXA-)scan (age ≥ 2 years), X-ray of the left hand (age ≤ 17 years), lower extremity MRI (age ≥ 10 years), accelerometry for 8 days (age ≥ 2 years), and questionnaires (patient report and/or parent proxy; age ≥ 2 years). All examinations are adapted to the patient’s age and functional abilities. Disease progression between all subsequent visits and relationships between outcome measures will be assessed.

**Discussion:**

This study will provide valuable insights into the 5-year natural history of patients with SELENON-RM and LAMA2-MD and contribute to further selecting relevant and sensitive to change clinical and functional outcome measures. Furthermore, this data will help optimize natural history data collection in clinical care and help develop clinical care guidelines.

**Trial registration:**

This study protocol including the patient information and consent forms has been approved by medical ethical reviewing committee (‘METC Oost-Nederland’; https://www.ccmo.nl/metcs/erkende-metcs/metc-oost-nederland, file number: 2023–16401). It is registered at ClinicalTrials.gov (NCT06132750; study registration date: 2023-10-05; study first passed date: 2023-11-15).

## Background

Selenoprotein N-related congenital myopathy (SELENON-RM) is a rare congenital myopathy with an estimated prevalence of 0.5 per 1,000,000 individuals [1]. The most common presenting sign is delayed motor development; other key features include slowly progressive axial muscle weakness, early-onset rigidity of the spine, scoliosis, and respiratory insufficiency [2]. Laminin-α2-related muscular dystrophy (LAMA2-MD) is a rare congenital muscular dystrophy with similar key features and an estimated prevalence of 8.3 per 1,000,000 individuals world-wide [3]. LAMA2-MD has a disease spectrum ranging from severe, early-onset congenital muscular dystrophy type 1A (MDC1A), to a milder childhood- or adult-onset limb-girdle-type muscular dystrophy. Additionally, patients may suffer from epileptic seizures and may show characteristic diffuse brain white matter lesions on magnetic resonance imaging (MRI) [4]. The clinical diagnosis of SELENON-RM and LAMA2-MD is confirmed by recessive pathogenetic loss-of-function variants in the LAMA2 or SELENON gene, respectively [5–9].

Given SELENON-RM and LAMA2-MD’s similar clinical phenotypes, they are well suited to be assessed in a basket natural history study [10, 11]. This can provide valuable insights into their similarities and differences. Currently, no curative treatment options exist for either SELENON-RM or LAMA2-MD; however, promising preclinical studies on novel therapeutic options are currently ongoing [12–21]. Therefore, obtaining natural history data is essential to reach trial readiness. To determine the effectiveness of potential treatment options in clinical trials, it is essential to identify and characterize patients clinically and genetically, in addition to selecting functional outcome measures that correlate with muscle function and are sensitive to temporal changes. For this purpose, the LAST STRONG study, a natural history study aiming to select outcome measures and reach trial readiness, was initiated in 2020 [22]. This study comprised a baseline visit and 1.5-year follow-up of SELENON-RM and LAMA2-MD patients, and the results demonstrated several interesting features of these disorders [10, 11, 23–25]. 90% of both SELENON-RM and LAMA2-MD patients had a low bone density irrespective of age. Despite initiating therapy to improve bone quality as needed, no changes in bone density were observed during the one-year follow-up period. Further, impaired respiratory function was observed in all SELENON-RM patients and in 85% of the LAMA2-MD patients. The SELENON-RM patients showed a progressive decline in respiratory function during 1.5-year follow-up. Additionally, cardiac involvement was observed in a subset of SELENON-RM and LAMA2-MD patients, implying that cardiac follow-up is desirable for optimal management. Based on these results, clear recommendations for clinical care for SELENON-RM and LAMA2-MD patients were formulated and locally implemented in clinical care [26].

Due to the slowly progressive character of SELENON-RM and LAMA2-MD, the LAST STRONG study only captured minor declining changes in functional abilities over the 1.5-year follow-up period. Therefore, we extended the natural history study with 3- and 5-year follow-up visits (Extended LAST STRONG). During a key-opinion leader (KOL) workshop in 2023 in Barcelona, experts from 14 countries gathered and discussed progress in the knowledge on natural history, pathophysiology, trial readiness, and potential treatment strategies for LAMA2-MD patients [27]. During this meeting, consensus was reached on harmonizing data collection protocols and defined a minimum dataset [27]. Based on the 1.5-year natural history study and the KOL, we selected a subset of tests for the Extended LAST STRONG study.

To summarize, the prospective 1.5-year natural history study with clinical and functional outcome measures (LAST STRONG) already gave promising outcomes [10, 11, 23–25]. With the Extended LAST STRONG study, we expect that extending this study to 3- and 5-year follow-up will increase knowledge on natural history of SELENON-RM and LAMA2-MD disease progression, and contribute to selecting relevant and selective clinical and functional outcome measures. Additionally, we aim to improve clinical care and international guidelines, and contribute to achieve trial readiness.

## Methods

### Study design

The Extended LAST STRONG (LAMA2-MD and SELENON-RM To Study Trial Readiness, Outcome measures and Natural history) is a single-center, prospective, observational study with repeated measurements performed at the Department of Neurology and Pediatric Neurology using the infrastructure of the neuromuscular center of expertise at Radboud university medical center, The Netherlands.

Patients will be invited for two outpatient visits, 3 and 5 years after the baseline visit of the LAST STRONG study. The Extended LAST STRONG study will expand to include patients that have newly diagnosed or patients who did not wish to participate in the previous study. In both visits patients will undergo a predefined subset of measurements (see Table 1) as previously established following the results of the LAST STRONG study and the KOL workshop [10, 11, 22–25, 27]. The study protocol was approved by the regional medical ethics committee and is registered on ClinicalTrials.gov (NCT06132750).

### Study population

No patient registry for LAMA2-MD and SELENON-RM currently exists in the Netherlands. Instead, we have aimed to identify all patients with LAMA2-MD and SELENON-RM mutations in the Netherlands and the Dutch-speaking part of Belgium through contact with genetic diagnostics services, rehabilitation centers, muscle disease experts, and patient organizations.

In the LAST STRONG study, 27 LAMA2-MD patients and 11 SELENON-RM patients were included. Given the high participation rate and minimal loss of follow-up during LAST STRONG, our expectation is to include a total of 35 to 40 patients in the extended study.

The inclusion criteria are identical to those used in the LAST STRONG study: first, a genetic confirmation of LAMA2-MD or SELENON-RM by two recessive pathological variants in the LAMA2 or SELENON gene, respectively, or typical clinical and histological characteristics combined with genetic confirmation in a first-degree relative; and second, patients should be willing and able to complete (part of) the measurement protocol at Radboud university medical center. If patients do not wish or are not able to visit our hospital, they will be offered the opportunity to participate in this study by sharing medical records, completing questionnaires, and undergoing a medical history and physical examination through home visits, video and/or telephone interviews. The only exclusion criterion is an insufficient understanding of the Dutch language.

### Recruitment

**Fig. 1:**
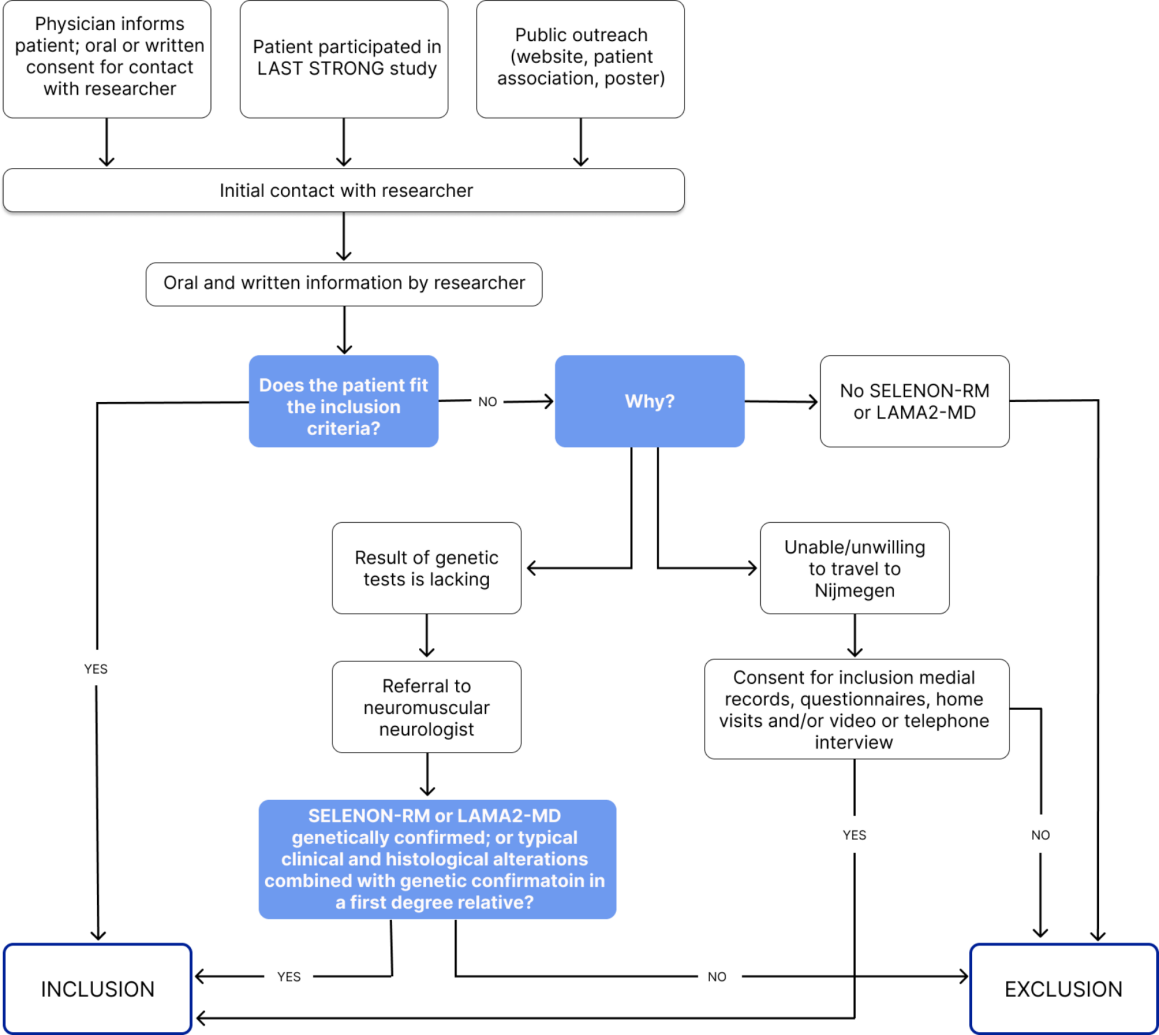
Flow-chart of recruitment and inclusion for the Extended LAST STRONG study

Participants will be consecutively recruited in the periods from October 2023 to September 2024 (see Fig. 1). Our recruitment strategy aims to include participants from both the pre-existing cohort of the LAST STRONG study and new participants. In addition, all patients known at our neuromuscular center will be personally informed. Further, we will directly recruit participants through promotion of our study on patient information days and through patient organizations.

### Demographics

Date of birth, sex, weight (kg), height (m), comorbidities, and medication will be recorded.

### Genetics

Upon enrollment in our study, all participants (or a first degree relative) will have undergone genetic examination as part of regular diagnostic work-up. For new participants, the genetic reports, including information on the specific genetic alterations, will be requested.

### Neurological examination and functional measurements

All participants will undergo a standard neurological examination performed by one assessor. Furthermore, muscle strength, facial muscle weakness, reflexes, muscle tone, and dysmorphic features will be assessed by two independent assessors. Muscle strength will be measured using the Medical Research Council (MRC) grading scale. The following muscles will be assessed: neck flexor, neck extensor, sternocleidomastoid, trapezius, deltoid, biceps brachii, triceps brachii, wrist extensor, wrist flexor, finger extensor, finger flexor, finger spreader, iliopsoas, gluteus maximus, quadriceps, hamstrings, foot dorsiflexor, foot plantarflexor, extensor hallucis longus and toe flexor. Additionally, muscle strength of the following muscles will be measured using a hand-held dynamometer and compared to normative values [28–31]:

1. Neck flexors and neck extensors: sitting upright; head up at 90 degrees from horizontal.

2. Elbow flexors and extensors: supine; shoulder adducted, elbow 90 degrees flexed, forearm supinated.

3. Knee extensors: sitting upright; knee 90 degrees flexed.

4. Foot plantar- and dorsiflexors: supine; foot 90 degrees dorsiflexed.

5. Pinch grip: sitting upright; shoulder adducted, elbow 90 degrees flexed, forearm pronated.

Furthermore, the passive range of motion (PROM) of the elbow, wrist, hip, knee, and ankle joints will be assessed by a goniometer [32].

A selection in functional outcome measures was made based on the outcomes of the LAST STRONG study and the KOL workshop, considering factors such as suitability, feasibility, low costs and reducing burden on participants. Functional measurements include [22]:

1. The Children’s Hospital of Philadelphia Infant Test of Neuromuscular Disorders (CHOP INTEND); age < 2 years [33, 34].

• The CHOP INTEND is a validated assessment of motor skills of children of 2 years and younger.

2. Hammersmith Infant Neurological Examinations (HINE); age < 2 years [35].

• The HINE is a simple and scorable method for evaluation of infants from 2 months to 2 years of age. It includes three sections that assess different aspects of neurological function, including neurological examination, developmental milestones and behavioral assessment.

3. Motor Function Measure – 20/32 (MFM-20/32); age ≥ 2 years [36, 37].

• In patients with neuromuscular diseases the motor function can be measured with the MFM-20/32. The MFM is a scale that consists of 20 or 32 items in three different dimensions. D1: standing position and transfers, D2: axial and proximal motor function, D3: distal motor function. MFM-32 is used in patients of 7 years and older. Children between the ages of 2 and 7 will undergo the MFM-20.

4. Graded and timed function tests; age ≥ 2 years: 30 s sit to stand, climb 4 stairs, descend 4 stairs, rise from floor; age ≥ 5 years: 6-Minute Walk test (6MWT) [38–41].

5. Functional Ambulation Classification (FAC); age ≥ 5 years [42].

• The FAC assesses functional ambulation in patients.

6. Brooke and Vignos Scale; age ≥ 2 years [43–45].

• The Brooke and Vignos scale provides ordinal-level data to assess upper- and lower-extremity function.

**Table 1:** Examinations performed in the Extended LAST STRONG study

Outcome domain	Assessments	Age
**Medical history**		
Past	Perinatal period, motor milestones	All
Current	Functional abilities, comorbidities, devices, treatments	All
**Neurological examination and functional measurements**		
Muscle function	Muscle strength assessment (Medical Research Council, MRC)	≥ 5 years
	Hand-Held dynamometry (HHD)	≥ 5 years
	The Children’s Hospital of Philadelphia Infant Test of Neuromuscular Disorders (CHOP INTEND)	< 2 years
	Hammersmith Infant Neurological Examinations (HINE)	< 2 years
	Motor Function Measurement (MFM)-20/32	≥ 2 years
	Graded and timed function tests; ambulant patients only 1. 30 s sit to stand, climb 4 stairs, rise from floor 2. 6-Minute Walk test (6MWT)	≥ 2 years ≥ 5 years
	Functional Ambulation Classification (FAC)	≥ 5 years
	Vignos and Brooke scale	≥ 2 years
Contractures	Goniometry	≥ 2 years
Other	Coordination, gait, reflexes, cranial and facial muscles, dysmorphic features	≥ 2 years
**Imaging**		
Muscles	Quantitative muscle ultrasound	All
	Quantitative muscle magnetic resonance imaging (MRI) of the lower extremities	≥ 10 years
Bone density	Dual-energy X-ray absorptiometry (DEXA)	≥ 2 years
	X-ray of the hand	2–17 years
**Respiratory assessment**		
Respiratory system	Spirometry (forced expiratory volume in the first second (FEV1), forced vital capacity (FVC), vital capacity (VC), peak cough flow (PCF))	≥ 5 years
	Maximum Expiratory Pressure (MEP), Maximum Inspiratory Pressure (MIP), Sniff Nasal Inspiratory Pressure (SNIP)	≥ 5 years
**Questionnaires**		
Activities and participation	ACTIVLIM	≥ 6 years
	Impact on Participation and Autonomy (IPA)	≥ 18 years
	Egen Klassifikation version 2 (EK2)	≥ 12 years
	Borg Rating Scale of Perceived Exertion; prior to and after 6MWT (Borg RPE)	≥ 5 years
Fatigue	Checklist Individual Strength (CIS)	≥ 18 years
	Pediatric Quality of Life Inventory (PedsQL) (Multidimensional Fatigue Scale; Child Self-report and/or parent proxy)	2 − 17 years
Pain	McGill pain questionnaire	≥ 12 years
	Wong-Baker Faces Pain rating scale	≥ 2 years
Quality of life	Pediatric Quality of Life Inventory (PedsQL) (Generic Core Scale, Neuromuscular Module; Child Self-report and/or parent proxy)	2–17 years
	Research and Development-36 (RAND-36)	≥ 18 years
	Individualized Neuromuscular Quality of Life (INQoL)	≥ 18 years
**Accelerometry**		
Accelerometry	Accelerometry using GENEActiv for eight consecutive days	≥ 2 years

### Imaging

#### Muscle ultrasound

At both visits, muscle thickness and muscle echogenicity (quantitative grayscale analysis) of the bilateral temporalis, sternocleidomastoid, deltoid, biceps brachii, flexor carpi radialis, flexor digitorum profundus, interosseus dorsalis I, rectus abdominis, rectus femoris, vastus lateralis, gastrocnemius caput medial, soleus and tibialis anterior will be assessed by muscle ultrasound using a Canon Aplio i800 with a PLT-1005BT Probe, following a strictly defined and fixed measurement protocol [46–54]. To measure muscle echogenicity, the mean grayscale level within a manually selected region of interest (ROI) in the ultrasound image will be calculated using an in-house developed software package in MATLAB (version R2022a, Mathworks, Natick, MA, USA). Muscle echogenicity and thickness will be standardized by calculating their z-score: [[measured grayscale level – predicted grayscale level] / standard deviation grayscale]. The predicted grayscale level is determined using a reference equation that includes age, height, sex, and weight [54, 55]. Further, all muscle ultrasound images will be visually evaluated using the semi-quantitative Heckmatt grading scale [56].

### Muscle MRI

During both visits, muscle features will be (semi-)quantitatively described through MRI (1.5 tesla, Siemens Healthineers, Erlangen, Germany) of the lower extremities in accordance with our locally developed scanning protocol, which includes chemical-shift-based water-fat-separation, or ‘Dixon’, imaging and T2 mapping. Both sequences are applied in four overlapping stacks, covering the upper and lower legs. The Dixon acquisition is a 3D gradient recalled echo scan with a voxel size of 0.9 mm x 0.9 mm x 3.0 mm, a repetition time (TR) of 6.9 ms, and two echo times (TE) of 2.4 and 4.8 ms—to obtain two sets of images where water and fat are in phase, and out of phase, respectively. Consistent with previous studies on (semi-)quantitative muscle assessment with MRI, the water and fat images derived from the Dixon in- and opposed-phase images will be used to generate a fat fraction map using MATLAB according to the following equation: Fat/(Fat + Water) [48]. Subsequently, the opposed-phase images will be used to draw ROIs per muscle using ITK-SNAP software. ROIs will be drawn at predetermined anatomical locations based on the localizer sequences. Each ROI will be checked by a second clinician. Muscle cross-sectional area and fat fractions will be calculated for each validated ROI. Our T2 mapping acquisition comprises a 2D multi-slice multi-echo spin-echo sequence with a voxel size of 3.9 mm x 3.9 mm x 12.0 mm, a TR of 2000 ms, and 17 TEs, ranging from 8 to 131 ms, to allow biexponential fitting of water and fat on a pixel-by-pixel basis [57]. The determined T2_water_ values, calculated over the same ROIs, can be used to quantitatively identify regions of edema.

### Bone density

In order to assess bone density, a Dual-energy X-ray absorptiometry (DEXA)-scan of the right femoral neck and lumbar spine, including a vertebral fracture assessment, will be performed using the Hologic Discovery A Horizon DXA System (S/N 303053 M). In pediatric patients (≤ 17 years of age), an additional X-ray of the hand will be performed to determine the Bone Health Index, in line with recommendations for clinical care in children with low bone quality.

### Respiratory function

Respiratory function will be assessed following the same protocol as implemented during the LAST STRONG study [22]. Patients aged 5 years and older will perform spirometry in upright and supine position (Pneumotrac, Spirotrac 6 Software, Vitalograph Inc., Maids Moreton, UK) during both visits. Additionally, patients will perform respiratory muscle strength testing to determine Maximum Expiratory Pressure (MEP), Maximum Inspiratory Pressure (MIP), and Sniff Nasal Inspiratory Pressure (SNIP) in an upright position (Pneumotrac, Spirotrac 6 Software, Vitalograph Inc., Maids Moreton, UK).

### Questionnaires

Patients and/or their parents will be asked to fill out standardized, age-adapted questionnaires on activities and participation, fatigue, pain, and quality of life, as follows.

1. ACTIVILIM; ≥ 6 years of age [58, 59].

• Questionnaire to assess the ability to perform 22 activities of daily life on a three-point scale, from impossible to easy.

2. Impact on Participation and Autonomy (IPA); ≥ 18 years of age [60].

• Questionnaire about participation and autonomy in daily life.

3. Egen Klassifikation version 2 (EK2); ≥ 12 years of age [61].

• Questionnaire that was designed to assess the functional ability of activity in daily living among non-ambulatory Duchenne muscular dystrophy patients. This questionnaire is available in English. Therefore, only patients who have a sufficient understanding of the English language will be asked to complete this questionnaire.

4. Borg Rating Scale of Perceived Exertion (Borg RPE scale); ≥ 5 years of age [62].

• Outcome measure that is used to assess physical activity intensity level. In the Extended LAST STRONG study participants are asked to assess their physical sensations prior to and post the 6MWT.

5. Checklist Individual Strength (CIS); ≥ 18 years of age [63, 64].

• Questionnaire that rates the following four subscales: subjective tiredness, concentration, motivation, and physical activation. It consists of 20 items on a seven-point scale.

6. McGill pain questionnaire; ≥ 12 years of age [65].

• Questionnaire that assesses the location, level, and characteristics of pain.

7. Wong-Baker Faces Pain rating scale; ≥ 2 years of age [66].

• The Wong-Baker Faces Pain rating scale is a visual analog scale, originally developed for children, consisting of a series of faces ranging from smiling to crying used to assess and communicate pain intensity.

8. The Pediatric Quality of Life inventory (PedsQL) (Generic Core Scale, Neuromuscular Module, Multidimensional Fatigue Scale; Child Self-report and/or parent proxy); pediatric patients 2–17 years [67–69].

• The PedsQL Generic Core Scale consists of 23 questions distributed across four domains: Physical, Emotional, Social, and School functioning. This scale has undergone translation and validation into various languages, including Dutch.

• The PedsQL Neuromuscular Module consists of 25 questions across three domains: Neuromuscular Disease, Communication, and Family Resources.

• The PedsQL Multidimensional Fatigue Scale evaluates subjective fatigue across three domains: General Fatigue, Sleep/Rest Fatigue, and Cognitive Fatigue.

9. The Research and Development-36 (RAND-36); ≥ 18 years of age [70].

• Questionnaire used to assess quality of life with 36 items across eight dimensions: physical functioning, role limitations due to physical health problems, role limitations due to emotional problems, energy/fatigue, emotional well-being, social functioning, pain, and general health perceptions.

10. The Individualized Neuromuscular Quality of Life (INQoL); ≥ 18 years of age [71].

• A measurement tool designed to evaluate quality of life specifically for individuals with neuromuscular disorders.

### Accelerometry

Patients (age ≥ 2 years) are asked to wear an accelerometer (GENEActiv Original, Activinsights Ltd) for 8 consecutive days after the study visit. Over the same time period, patients or their parents are asked to fill in a diary with their major activities, including sleeping hours, physical exercise, work, and school. The GENEActiv is a tri-axial accelerometer worn on the wrist that will be configured to record data at a sampling rate of 87.5 Hz [72–74]. Subsequently, the raw data will be processed into 1-second epochs by using the GENEActiv Software (v.3.3, 2019). We will use the gravity subtracted sum of vector magnitudes (SVMgs) as the activity measure. The SVMgs will be calculated using the following equation: SVMgs = ∑√(x^2^+γ^2^+z^2^)-1g. All data will be analyzed using MATLAB R2022a for windows. A large subset of variables will be analyzed, including total activity (counts/day) and the percentage of sedentary, light, moderate, and vigorous activity.

### Neurocognitive functioning

To characterize central nervous system manifestations in patients with SELENON-RM and LAMA2-MD, all cerebral MRI scans that were previously conducted in standard clinical care will be collected and re-assessed for structural abnormalities; if they have not been conducted before, they will not be requested. Furthermore, information on neurocognitive function (education, grade retention, job, social functioning, and participation) will be collected retrospectively from medical history surveys during the natural history study.

### Statistical methods

We will use descriptive statistics to summarize our data. To evaluate disease progression between the follow-up visits of the LAST STRONG and Extended LAST STRONG, mixed-models will be applied. Multiple linear regressions will be used for the exploration of relationships between potential disease modifying variables and disease severity. To investigate relationships between outcome measures, Pearson correlation analysis will be performed. For comparing parameters (e.g. MFM-20/32, accelerometry, bone quality) between ambulant and non-ambulant patients, independent sample t-tests will be performed. Statistical significance will be considered at *p* < 0.05.

### Data collection

All data-management and data-monitoring will be performed using Castor CDMS (version 2024.1.3.0) through direct and indirect entry via our electronic patient system (Epic, version May 2023). Raw data such as muscle ultrasound images, accelerometry data and MRI images will be stored on a department server in a secured folder. All data will be available from the corresponding author on reasonable request.

### Study scheduling

Patients will be included between October 2023 and October 2024 and will be invited to return after 2 years (October 2025 – October 2026). The last visit of the last included patient is expected to take place in October 2026.

## Discussion

We present the design of the Extended LAST STRONG study, a long-term natural history study in a representative cohort of Dutch-speaking SELENON-RM and LAMA2-MD patients, including both adults and children. By extending the study to a 5-year follow-up, we are able to gain a more comprehensive understanding on longer-term disease progression across the whole lifespan and contribute to identifying relevant and selective clinical and functional outcome measures. This study includes an extensive set of clinical and functional outcome measures, enabling us to assess the clinical spectrum of patients diagnosed with SELENON-RM and LAMA2-MD. Our primary aims are: (1) to collect 3- and 5-year natural history data in patients with SELENON-RM and LAMA2-MD; and (2) to select relevant and sensitive clinical and functional outcome measures to achieve trial readiness. Additionally, these insights will be needed to reach implementing collection of natural history data into clinical care and international guidelines.

Major strengths of this study are the inclusion of a wide range in participants’ age (both pediatric and adult patients) and disease severity, a plethora of outcome measures, and the prospective data collection. Unlike other natural history studies that have focused mainly on pediatric cohorts, this study will provide insights into the long-term natural history of these two rare neuromuscular diseases across the lifespan.

Following the outcomes of the LAST STRONG study and the KOL workshop, a subset of qualitative and quantitative measurements was selected for the Extended LAST STRONG study, tailored to the age and functional abilities of the participants [10, 11, 23–25, 27]. Baseline findings on the functional outcome measures of the LAST STRONG indicated that the Hammersmith Functional Motor Scale, Pediatric Balance Scale, and Mini Balance Evaluation Systems Test were neither suitable nor feasible for evaluation disease progression and/or severity in both SELENON-RM and LAMA2-MD. Therefore, these outcome measures are not performed in the Extended LAST STRONG study. Research in other neuromuscular disorders has shown that the MFM-20/32, graded timed function tests and 6MWT are possible relevant and sensitive clinical and functional outcome measures [75–79]. Because of the slowly progressive nature of SELENON-RM and LAMA2-MD, only small changes in functional abilities were observed. Therefore, these outcome measures should be further investigated. Recent research in facioscapulohumeral muscular dystrophy observed significant correlations between muscle imaging techniques as MRI and muscle ultrasound and changes in clinical outcome measures [53, 80, 81]. Previous natural history studies on SELENON-RM and LAMA2-MD did not include muscle imaging biomarkers. Since our hospital is a recognized neuromuscular imaging center of expertise and has experience with the applicability of imaging in natural history studies, it is expected to identify novel muscle biomarkers in SELENON-RM and LAMA2-MD.

The importance of trial readiness is explained by the development of promising new treatment strategies for SELENON-RM and LAMA2-MD [12–21]. For LAMA2-MD, novel therapeutic approaches—divided into genetic specific and non-specific strategies—are being investigated, whereas in SELENON-RM, only genetic non-specific approaches are being investigated. Internationally, natural history and outcome measure studies for patients with LAMA2-MD are currently ongoing in France, Spain, Switzerland, Italy, Brazil, the United States, and the Netherlands. The ambition of the Lama2-Europe initiative, a collaborative network focused on LAMA2-MD, is to extend these studies to additional countries, using the LAST STRONG study protocol as a leading example, to enhance both the scope and impact of the research [27].

We are working towards the implementation of data collection into routine clinical care. This approach combines scientific research with clinical practice, allowing patients to undergo examinations only once, which is more efficient and patient friendly. During the KOL workshop, a minimal clinical dataset, including the CHOP INTEND (< 2 years of age) and/or the MFM-32/20 (> 2 years of age), motor function, range of motion of elbow and knee joints, rigid neck, adherence to standards of care, passive range of motion, and photogrammetry for monitoring respiratory function, was proposed [27]. However, a definitive agreement on a minimal dataset is yet to be reached and will be established through regular online meetings of an international consortium of clinicians and researchers.

Given that specific guidelines for standardizing and optimizing clinical care for patients are still lacking, and the existing general clinical care guidelines for congenital muscular dystrophies are outdated [82], an ENMC workshop for LAMA2-MD is scheduled for January 2025 [83]. This workshop aims to establish specific clinical care guidelines, with one of the focus areas being bone health, following the recent findings of the LAST STRONG study. The process established for developing these clinical care guidelines is intended to serve as a model for other congenital muscular disorders subtype-specific guidelines. These guidelines will ensure that all patients receive the same standard of care, which is important for possible future clinical trials. The ability of these trials to evaluate the potential efficacy of a therapeutic intervention will depend on all trial participants receiving the same standard of care at the time of enrollment.

Furthermore, after gathering all long-term natural history data we aim to enter all data from the cohort in the EURO-NMD registry hub [84]. Currently, there are no unified registries for SELENON-RM and LAMA2-MD within the European Union. For future studies, we recommend entering participant data in the EURO-NMD registry hub to ensure interoperability.

In our study, patients are invited to participate in the Radboud Biobank [85] for storage of blood and urine samples in accordance with the general biobank protocol [86]. In future research, these samples can be analyzed for biomarkers. Further, muscle biopsy and staining should be further investigated since it would provide crucial information for further genotype/phenotype correlations, especially for the common Dutch mutation.

## Conclusion

The Extended LAST STRONG study is expected to provide comprehensive long-term natural history data, which can be used to further select relevant clinical and functional outcome measures to achieve trial readiness in SELENON-RM and LAMA2-MD patients. Further, our study aims to implement collection of natural history data into clinical care and contribute to the development of international care guidelines.

## Data Availability

The datasets generated during the current study will be available in the Donders Respiratory (https://data.donders.ru.nl).

## Abbreviations

Borg RPE: Borg Rating of Perceived Exertion scale
CHOP INTEND: The Children’s Hospital of Philadelphia Infant Test of Neuromuscular Disorders
CIS: Checklist Individual Strength
DEXA: Dual-energy X-ray absorptiometry
EK2: Egen Klassification version 2
ENMC: European Neuromuscular Centre
EURO-NMD: European Reference Network for neuromuscular diseases
FAC: Functional Ambulation Classification
FEV1: Forced expiratory volume in the first second
FVC: forced vital capacity
HHD: Hand-held dynamometry
HINE: Hammersmith Infant Neurological Examinations
INQoL: Individualized Neuromuscular Quality of Life
IPA: Impact on Participation and Autonomy
KOL: key-opinion leader
LAMA2-MD: Laminin-a2-related muscular dystrophy
MDC1A: Merosin-deficient congenital muscular dystrophy type 1 A
MEP: Maximal Expiratory Pressure
MFM-20/32: Motor function measure – 20/32
MIP: Maximal Inspiratory Pressure
MRI: Magnetic resonance imaging
MRC: Medical Research Council
PCF: peak cough flow
PedsQL: Pediatric Quality of Life Inventory
PROM: Passive range of motion
RAND-36: Research and Development-36
ROI: Region of interest
SELENON-RM: SELENON-related myopathy
SNIP: Sniff Nasal Inspiratory Pressure
SVMgs: The gravity of subtracted sum of vector magnitudes
VC: Vital capacity
6MWT: 6-Minute Walk test
