# Correction: A 5-year natural history study in LAMA2-related muscular dystrophy and SELENON-related myopathy: the Extended LAST STRONG study

**DOI:** 10.1186/s12883-024-03994-5

**Published:** 2024-12-16

**Authors:** E. C. M. de Laat, S.L.S. Houwen- van Opstal, K. Bouman, J. L. M. van Doorn, D. Cameron, N. van Alfen, A. T. M. Dittrich, E. J. Kamsteeg, H. J. M. Smeets, J. T. Groothuis, C. E. Erasmus, Nicol C. Voermans

**Affiliations:** 1 Department of Neurology, Donders Institute for Brain, Cognition and Behaviour, Radboud University Medical Center, Nijmegen, The Netherlands; 2 Department of Rehabilitation, Donders Institute for Brain, Cognition and Behaviour, Radboud University Medical Center, Amalia Children’s Hospital, Nijmegen, The Netherlands; 3 Department of Neurology, Clinical Neuromuscular Imaging Group, Donders Institute for Brain, Cognition and Behaviour, Radboud University Medical Center, Nijmegen, The Netherlands; 4 Department of Radiology, Clinical Neuromuscular Imaging Group, Radboud University Medical Center, Nijmegen, The Netherlands; 5 Department of Pediatrics, Radboud University Medical Center, Radboud Institute for Health Sciences, Amalia Children’s Hospital, Nijmegen, The Netherlands; 6 Department of Human Genetics, Radboud University Medical Center, Nijmegen, The Netherlands; 7 Department of Toxicogenomics, Research Institutes MHeNS and GROW, Maastricht University, Maastricht, The Netherlands; 8 Department of Pediatric Neurology, Donders Institute for Brain, Cognition and Behaviour, Amalia Children’s Hospital, Radboud University Medical Center, Nijmegen, The Netherlands

**Correction: BMC Neurol 24**,** 409 (2024)**

10.1186/s12883-024-03852-4

Following publication of the original article [1], the authors reported that the last author’s name “Nicol C. Voermans” has been repeated twice in the author group. The duplicate author name has been removed.

The original article [1] has been updated.
